# Methodology and Results of a Joint IFSO-WGO Delphi Survey of 94 Intercontinental, Interdisciplinary Experts in Obesity Management

**DOI:** 10.1007/s11695-023-06757-2

**Published:** 2023-10-13

**Authors:** Lilian Kow, Reem Z. Sharaiha, Mary O’Kane, Kevin P. White, Guilherme Macedo, Jim Toouli, Scott Shikora

**Affiliations:** 1 Dept. of Surgery, Flinders University, Adelaide, SA Australia; 2 Dept. of Gastroenterology, Weill Cornell Medical College, New York, NY USA; 3 Leeds Teaching Hospitals NHS Trust, Leeds, UK; 4 ScienceRight International Health Research Consulting (SRIHRC), London, ON Canada; 5 Dept. of Gastroenterology & Hepatology, Centro Hospitalar de São João, Porto, Portugal; 6 Center for Metabolic and Bariatric Surgery, Brigham and Women’s Hospital Harvard Medical School, Boston, MA USA

**Keywords:** Obesity, Management, Treatment, Bariatric surgery, Bariatric endoscopy, Delphi survey, Consensus

## Abstract

**Background:**

Obesity affects 1.5 billion people worldwide, yet few are treated effectively and considerable variability exists in its management. In 2020, a joint International Federation of Surgery for Obesity and Metabolic Diseases (IFSO) and World Gastroenterology Organization (WGO) advisory committee initiated the drafting of consensus guidelines on obesity management, to be based on detailed literature reviews and the results of an extensive multi-disciplinary survey of intercontinental experts. This paper reports on the latter. The objective of this study is to identify areas of consensus and non-consensus among intercontinental, inter-disciplinary experts in obesity management.

**Methods:**

Guided by an international consensus-survey expert, a three-round online Delphi survey was conducted in the summer of 2021 of international obesity-management experts spanning the fields of medicine, bariatric endoscopy and surgery, psychology, and nutrition. Issues like epidemiology and risk factors, patient selection for metabolic and bariatric surgery (ASMBS-Clinical-Issues-Committee, Surg Obes Relat Dis : Off J Am Soc Bariatric Surg. 8:e27-32, 1), psychological issues, patient preparation for MBS, bariatric endoscopy, and outcomes and follow-up were addressed.

**Results:**

Ninety-four experts from six continents voted on 180 statements, with consensus reached on 158, including consensus agreement with 96 and disagreement with 24 statements (38 had other response options besides agree/disagree). Among unanimous opinions were the need for all medical societies to work together to address obesity, for regular regional and national obesity surveillance, for multi-disciplinary management, to recognize the increasing impact of childhood and adolescent obesity, to accept some weight regain as normal after MBS, and for life-long follow-up of MBS patients.

**Conclusions:**

Obesity is a major health issue that requires aggressive surveillance and thoughtful multidisciplinary management.

## Introduction

Approximately 1.5 billion people worldwide currently live with obesity [1], and this number is rising [2–4], even among children and adolescents [5]. Beyond its own implications for health and fitness, obesity increases the risk of numerous other potentially life-threatening complications, like type 2 diabetes mellitus (T2DM) [6], cardiovascular disease [7], and at least 13 distinct types of cancer [8, 9]. Excess weight has also been linked to significantly decreased quality of life (QoL) [2], significantly increased risk of early mortality, decreased life expectancy [10], and increased cancer-related mortality [11]. These risks even extend to childhood obesity [12].

Managing obesity is difficult, with “eating less and exercising more” rarely attaining long-term success. Consequently, and because of the numerous obesity-associated comorbidities, obesity has been termed “a chronic relapsing progressive disease” [13]. While dietary changes, other lifestyle changes like exercise, and counselling remain the first line of treatment, relatively recent advances in obesity management have included pharmacological, endoscopic, and surgical interventions. Metabolic and bariatric surgery (MBS) remains significantly more effective than dietary and lifestyle changes alone at inducing weight loss, reducing comorbidities, and improving QoL [14–17].

Several operative interventions currently exist and which procedure is chosen and when to offer surgery varies between practices and regions [18]. Bariatric procedures also carry their own risks, including a low, but non-negligible (0.15–0.35%) risk of peri-operative mortality [19, 20]. Additional complications of MBS include potentially fatal nutritional deficiencies [21–24]; post-operative bleeding, intestinal obstructions, severe gastroesophageal reflux, and various gastrointestinal syndromes [19]. Patients undergoing MBS may also be prone to developing new post-operative addictive behaviours like substance abuse [25]. Consequently, MBS should not be used to replace, but to supplement other, non-operative approaches to obesity management, including dietary and lifestyle changes. It is also important to identify and treat psychopathology, utilizing psychosocial counselling and pharmacotherapy [25]. However, like choosing operative procedures, variability exists in how and to what extent such services are co-administered [26]. Variability also exists in which patients are considered eligible and safe for endoscopic and bariatric procedures [27]; how to define treatment success and failure [16, 28]; how much weight regain should be considered acceptable [29]; and which metric to utilize for measuring weight regain (e.g., as a percentage of excess vs. total weight) [30].

It was such variability and uncertainty that led the World Gastroenterology Organization (WGO) and International Federation for the Surgery of Obesity and Metabolic Disorders (IFSO) to join forces in 2020 to take steps towards drafting international guidelines on the management and long-term monitoring of obesity. This included undertaking a survey of 94 international, interdisciplinary experts in obesity management to identify areas of consensus and non-consensus spanning a range of topics. This paper reports those results.

## Methods

An online modified Delphi survey was conducted following published guidelines [31]. The Delphi approach was adopted because of its exponentiallyincreasing utilization in health science and other fields and its unanimous voting, thereby reducing the risk of conformity/acquiescence bias typically ascribed to in-person consensus meetings [31].

Survey development with each steering committee member generating a list of issues/questions of major interest within their own discipline. To be considered for survey inclusion, the issue could not already be considered a firmly established, universal standard of care based upon published empirical evidence, but had to be considered of appreciable importance to obesity management. Issues of interest ranged from epidemiology and public perceptions to treatment and follow-up.

All submitted statements were sent to the steering committee for statement selection; then to the Delphi expert for editing, consolidation into a single survey, and reformatting to ensure comprehensibility and consistency. Several steps were undertaken to reduce any risk of bias potentially induced by the survey itself, including primarily using non-judgmental statements (e.g., neither favouring nor opposing a particular concept/belief/approach); balancing the remaining favourable and unfavorable statements; and altering the order of response options to minimize order bias (e.g., favorable options listed anywhere from first to last). The survey’s first full draft was sent to all steering-committee members for feedback and potential modification, after which a pilot survey of 10 experts was conducted to identify all language, factual, or conceptual issues.

The final Round 1 survey had 157 statements subdivided into six modules: *Module 1*: Epidemiology and risk factors (20 statements); *Module 2*: Patient selection for MBS (29 statements); *Module 3*: Psychological issues (14 statements); *Module 4*: Patient preparation for MBS (23 statements); *Module 5*: Bariatric endoscopy (39 statements, only voted on by surgeons and endoscopists); and *Module 6*: Outcomes and follow-up (32 statements). Statements failing to achieve ≥ 70% consensus were included in a second-round survey. Each expert was asked at both the start and end of each module how comfortable they felt voting on that module’s focus, rated from very uncomfortable to very comfortable so votes from uncomfortable voters could be excluded during data analysis. At least 80% participation of eligible voters on any statement was required for that statement’s vote tally to be considered valid.

In June 2021, an email was sent to 100 experts who had previously agreed to participate in the survey, including a link to the above-mentioned, committee-approved Round 1 survey on the online platform Survey Monkey. Practice characteristics of the 94 who ultimately participated, and of the *n* = 37 bariatric surgeons and *n* = 55 with experience in bariatric endoscopy are summarized in Table 1.

**Table 1:** Practice characteristics of the expert panel

	*N* =	Percentage of total
Continent		
Africa	2	2.1%
Asia	15	16.0%
Europe	26	27.7%
Latin America	10	10.6%
Middle East	7	7.4%
North America	28	29.8%
Oceania	6	6.4%
Total	94	100.0%
Specialty		
Bariatric endoscopy	18	19.1%
Bariatric surgery	37	39.4%
General medicine	6	6.4%
Hepatology	15	16.0%
Psychology	4	4.3%
Nutrition	14	14.9%
Total	94	100.0%
Nature of clinical practice		
Primarily university based	59	62.8%
Some university affiliation	25	26.6%
Non-academic	10	10.6%
Total	94	100.0%
Member of obesity care team		
Yes	85	90.4%
No	9	9.6%
Total	94	100.0%
% Time managing patients with obesity		
< 25%	21	22.3%
25–50%	26	27.7%
> 50%	47	50.0%
Total	94	100.0%
Years managing patients with obesity		
< 5 years	5	5.3%
5–10 years	18	19.1%
> 10 years	71	75.5%
Total	94	100.0%
Years performing bariatric procedures		
< 5 years	7	12.7%
5–10 years	10	18.2%
> 10 years	38	69.1%
Total	55	100.0%
Surgeons only (*N* = 37)		
Minimally invasive surgery only	27	73.0%
Open surgery only	0	0.0%
Both minimally invasive and open surgery	10	27.0%
Total (Surgeons only)	37	100.0%
Surgeons and endoscopists (*N* = 55)		
Roux-en-Y bypass	41	74.5%
Sleeve gastrectomy	42	76.4%
MGB-OAGB	18	32.7%
Other	39	70.9%
Intra-gastric balloon placement	35	63.6%
ESG	20	36.4%
POSE	5	9.1%
Aspiration therapy	7	12.7%
Other	14	25.5%

*MGB-OAGB* mini gastric bypass, also called one anastomosis gastric bypass, *ESG* endoscopic sleeve gastroplasty, *POSE* primary obesity surgery using an endoluminal approach

## Results

Among the five modules open to all experts, voter numbers ranged from 80 to 94 (85–100%) out of 94; for Module 5, restricted to 58 bariatric surgeons and/or endoscopists, voter numbers ranged from 54 to 58 (94.7–100%). Hence, a valid vote was achieved for every statement.

After Round 1 results were analyzed, 23 statements on the “relative importance of pre-operative patient factors” were added to the Round 2 survey. Final analysis was, therefore, of 180 (157 + 23) statements.

Among the 180 statements included in final analysis, only 17 (9.4%) were deemed by the advisory panel as favorable to a particular concept/belief/approach, 19 unfavorable (10.6%), and 144 (80.0%) non-judgmental (Table 2). An abbreviated third round of voting was conducted for the eight of 23 statements added to Round 2 for which no consensus was achieved in that round, thereby permitting two rounds of voting on all statements requiring a second vote.

**Table 2:** Overall summary of results over two rounds of voting

Statements	*N* =	% =
Total number of statements	180	100%
*Consensus reached*	*158*	*87.8%*
*No consensus reached*	*22*	*12.2%*
Consensus reached in 1st round	114	72.2%
Consensus reached in 2nd round	44	27.8%
% Statements consensus reached—Epidemiology and risk factors (20 statements)	18	90.0%
% Statements consensus reached—Patient selection (29 statements)	24	82.8%
% Statements consensus reached—Relative importance of pre-op factors (23 factors)	21	91.3%
% Statements consensus reached—Psychological issues (14 statements)	12	85.7%
% Statements consensus reached—Patient preparation-general (10 statements)	9	90.0%
% Statements consensus reached—Patient preparation-COVID-19 (13 statements)	13	100.0%
% Statements consensus reached—Bariatric endoscopy (39 statements)	31	79.5%
% Statements consensus reached—Outcomes and follow-up (32 statements)	30	93.8%
100% consensus reached	12	7.6%
90–99% consensus reached	43	27.2%
80–89% consensus reached	68	43.0%
70–79% consensus reached	35	22.2%
Statements agreed with (total)	104	57.8%
Statements disagreed with (total)	30	16.7%
Statements agreed with (consensus)	96	60.8%
Statements disagreed with (consensus)	24	15.2%
*Statements worded favorably to bariatric interventions*	*17*	*9.4%*
*Statements worded unfavorably to bariatric interventions*	*19*	*10.6%*
*Non-judgmental statements*	*144*	*80.0%*
Average consensus—Epidemiology and risk factors	84.7%	
Average consensus—Patient selection	84.3%	
Average consensus—Relative importance of pre-op factors	86.5%	
Average consensus—Psychological issues	81.3%	
Average consensus—Patient preparation-general	84.6%	
Average consensus—Patient preparation-COVID-19	82.8%	
Average consensus—Bariatric endoscopy	78.0%	
Average consensus—Outcomes and follow-up	87.9%	
Average consensus—Overall	83.6%	
*Minimum/maximum level of consensus on a statement*	*50%/100%*	
*Min. when consensus reached*	*70.5%*	

At least 70% consensus was achieved on 158 statements (87.8%)—114 first round, 44 s round. However, 100% consensus was only achieved for 12 statements. Table 2 provides further general results.

The results for each of the six modules are summarized individually in Tables 3, 4, 5, 6, 7, 8, and 9, with Module 2 subdivided into part A (Table 4) and part B (Table 5).

**Table 3:** Module 1—Epidemiology and risk factors (*N* = 94 voters in round 1; 79 in round 2)

Statements (*N* = 20)	N*	Rounds required	Most common selection	Percent consensus
Since obesity is a major contributor to the global burden of chronic disease, disability, and healthcare costs, all medical societies should cooperate to address this problem systematically	94	1	Agree	100.0%
Longitudinal national and regional surveillance of obesity, with measured data, should be conducted on a regular basis	94	1	Agree	100.0%
Obesity is a chronic disease, caused by abnormal or excess body fat accumulation that impairs health and increases the risk of premature morbidity and mortality	94	1	Agree	97.9%
Emotional eating is a common feature of obesity	94	1	Agree	97.9%
Ethnicity and geographical origins are important factors in the pathophysiology of obesity and metabolic diseases	94	1	Agree	91.5%
Interventions for obesity and metabolic diseases should take the patient’s ethnicity and geographic location into consideration	94	1	Agree	90.4%
There are individuals who, despite being severely obese, never experience eating binges	94	1	Agree	90.4%
Food addiction cannot exist, since food contains no substances capable of acting directly on brain areas related to reward processing	91	1	Disagree	87.9%
All individuals with obesity have eating binges	94	1	Disagree	85.1%
Emotional eating and food addiction are the most common causes of eating binges in candidates for bariatric surgery	79	2	Agree	84.8%
Some patients with problematic alcohol use pre-operatively exhibit less problematic alcohol use after they undergo bariatric surgery	79	2	Disagree	84.8%
Patients addicted to food develop alcohol or other substance abuse after bariatric surgery	79	2	In a minority of cases	83.5%
Candidates for bariatric surgery with a history of binge eating are more prone to experience undesirable behavioral outcomes after bariatric surgery than candidates with no history of binge eating	94	1	Agree	81.9%
Food addiction is a common feature of obesity	79	2	Agree	81.0%
Sufficient empirical evidence exists to consider “food addiction” a valid clinical entity	79	2	Agree	79.7%
Food addiction is more common in candidates for bariatric surgery who exhibit problematic use of alcohol or other mood-altering substances	75	2	Agree	78.7%
Candidates for bariatric surgery with a history of binge eating are more prone to suicide or suicidal behaviors after bariatric surgery than candidates with no history of binge eating	79	2	Disagree	77.2%
Candidates for bariatric surgery with a history of binge eating are more prone to regain weight after bariatric surgery than candidates with no history of binge eating	93	1	Agree	76.3%
*Emotional eating is more common in candidates for bariatric surgery than in other people who are obese*	*79*	*2*	*Disagree*	*68.4%*
*The great majority of candidates for bariatric surgery have an addiction to food*	*79*	*2*	*Disagree*	*55.7%*

Italicized statements did not reach consensus

*N = number of voters in the final/definitive round of voting on the statement

**Table 4:** Module 2 (part A)—Patient selection (*N* = 94 voters in round 1; 79 in round 2)

Statements (N = 29)	N*	Rounds required	Most common selection	Percent consensus
Global rates of obesity are currently increasing in children and adolescents	94	1	Agree	100.0%
Most children and adolescents with obesity grow up to have obesity in adulthood	93	1	Agree	100.0%
Children and adolescents with severe obesity are at risk of significant obesity-related comorbidities, like type 2 diabetes mellitus, hypertension, etc	94	1	Agree	100.0%
Metabolic and bariatric surgery in adolescents requires a multidisciplinary team [e.g., pediatric psychologists and endocrinologists] with experience dealing with children & adolescents & their families	93	1	Agree	100.0%
Lack of physician and public knowledge, as well as the lack of long-term results of MBS in adolescents, represent some of the potential barriers for referral of adolescents for MBS	92	1	Agree	100.0%
Life-long follow up is needed in adolescents who undergo metabolic bariatric surgery [1]	92	1	Agree	98.9%
Bariatric surgery in the elderly improves their overall quality of life (QoL)	90	1	Agree	96.7%
In adolescents, MBS should be performed by experienced bariatric surgeons with a proven track record performing MBS in adults	91	1	Agree	95.6%
Short-term studies show that MBS in adolescents is safe and leads to excellent outcomes, including durable weight loss and improvements in obesity-related medical problems and quality of life	89	1	Agree	95.5%
Life span expectations should be taken into account when considering bariatric surgery for elderly patients	92	1	Agree	90.2%
Sleeve gastrectomy is the most common procedure performed in adolescents, followed by Roux-en-Y gastric bypass	87	1	Agree	89.7%
The choice between sleeve gastrectomy and Roux-en-Y gastric bypass in adolescents should be based on BMI, and the presence versus absence of comorbidities like GERD and diabetes	87	1	Agree	88.5%
Besides the extent of obesity and the patient’s consent, a patient’s age should be the only consideration when surgeons are planning bariatric surgery in an elderly	94	1	Disagree	87.2%
Laparoscopic Roux-en-Y gastric bypass (LRYGB) should be considered a viable option for patients who are elderly	91	1	Agree	86.8%
The 30-day post-operative mortality risk of 0.4% in patients over 65 years (versus 0.1% in younger patients) contraindicates bariatric surgery in this patient group	89	1	Disagree	86.5%
The amount of weight loss achieved should *not* be the primary indicator of treatment success in patients who are elderly	94	1	Agree	86.2%
Short-term studies show that MBS in adolescents is similar to MBS in adults, in terms of major complications, readmissions, and mortality	86	1	Agree	86.0%
Biliopancreatic diversion [duodenal switch] and one anastomosis gastric bypass are not recommended in adolescents	87	1	Agree	85.1%
Operating time directly impacts the rate of complications in the elderly	86	1	Agree	83.7%
Only high-volume bariatric services and experienced bariatric surgeons should operate on patients who are elderly	91	1	Agree	82.4%
Enough empirical evidence has been published to affirm that metabolic and bariatric surgery [1] is the most effective therapy for severe obesity in adolescents	92	1	Agree	79.3%
The overall risk of bariatric surgery may be prohibitive in patients who are elderly	79	2	Disagree	77.2%
The rate of obesity in adolescents is increasing without a similar increase in the rate of adolescent MBS	90	1	Agree	71.1%
Peri-operative risk in the elderly is comparable to that of younger patients	93	1	Disagree	71.0%
*Patients who are elderly can undergo hypo-absorptive procedures*	*79*	*2*	*Agree*	*69.6%*
*In terms of weight loss, patients who are elderly tend to respond more, less, or about the same to a laparoscopic Roux-en-Y gastric bypass (LRYGB) than patients who are younger*	*79*	*2*	*About the same*	*65.8%*
*In terms of weight loss, patients who are elderly tend to respond more, less, or about the same to a laparoscopic sleeve gastrectomy (LSG) than patients who are younger*	*79*	*2*	*About the same*	*60.8%*
*For elderly patients with metabolic syndrome, the gold standard procedure should be… (LRYGB, LSG, other)*	*78*	*2*	*LRYGB*	*60.3%*
*In terms of bariatric surgery, a patient should start to be considered elderly…*	*79*	*2*	*Based on physiological age*	*51.3%*

Italicized statements did not reach consensus

*BMI* body mass index, *MBS* metabolic and bariatric surgery, *LRYGB* laparoscopic Roux-en-Y gastric bypass, *LSG* laparoscopic sleeve gastrectomy, *GERD* gastroesophageal reflux disease

*N = number of voters in the final/definitive round of voting on the statement

**Table 5:** Module 2 (part B)—Relative importance of pre-operative patient factors (*N* = 79 voters in round 2)

Statements (*N* = 23)	*N*	Level of importance	Percentage consensus
Patient's levels of general health and fitness	79	Very	98.7%
The presence and/or nature of comorbid illness	79	Very	97.5%
Ability to understand/cognitive level	79	Very	96.2%
Alcohol or other substance abuse	79	Very	96.2%
Psychological health and illness	79	Very	94.9%
Cardiovascular health	79	Very	94.9%
Liver health (including cirrhosis and portal hypertension)	78	Very	94.9%
Patient's level of compliance	79	Very	92.4%
Obesity's impact on patient’s quality of life	79	Very	92.4%
Patient's nutritional status	79	Very	91.1%
Physiological more than chronological age	79	Very	89.9%
Kidney function	78	Very	89.7%
Respiratory health	79	Very	88.6%
Social and/or family network and support	79	Very	84.8%
Presence/nature of physical disabilities	79	Very	84.8%
Current smoking status	79	Very	84.8%
Advanced diabetes mellitus	79	Very	83.5%
Muscle mass (risk of sarcopenia)	78	Very	83.3%
Life span expectations	79	Very	82.3%
Patient's level of physical mobility	79	Very	81.0%
Bone health	79	Very	73.4%
*Financial means (e.g., ability to afford vitamins)*	*79*	*Very*	*59.5%*
*Thyroid disease*	*78*	*Not very*	*53.8%*

This list was added in response to an open-ended question asking voters to list factors they considered important in the decision to perform and how to perform surgical or endoscopic bariatric interventions. The order of factors is from highest to lowest percentage perceiving a factor as important. Italicized statements did not reach consensus

**Table 6:** Module 3—Psychological issues (*N* = 94 voters in round 1; 79 in round 2)

Statements (*N* = 14)	N*	Rounds required	Most common selection	Percentage consensus
Patients undergoing bariatric surgery virtually always develop problematic alcohol use post-operatively	91	1	Disagree	95.6%
Patients with severe psychiatric conditions, like schizophrenia or bipolar disorder, should not undergo bariatric surgery, unless the psychiatric condition is well controlled	91	1	Agree	95.6%
A comprehensive psychological evaluation should be completed before bariatric surgery	94	1	Agree	93.6%
Candidates for MBS with predominantly cognitive depressive symptoms (e.g., difficulty concentrating, memory loss) usually do not exhibit any improvement in their depressive symptoms after surgery	78	2	Disagree	89.7%
Most patients with depression experience worsening of their depressive symptoms after bariatric surgery	88	1	Disagree	87.5%
Candidates for bariatric surgery who predominantly have somatic depressive symptoms—like asthenia, fatigue, and psychomotor retardation—tend to be less depressed after bariatric surgery	79	2	Agree	84.6%
The best psychotherapeutic strategy for patients with obesity and a high risk of binge eating behavior is…	86	1	CBT	83.7%
Candidates for bariatric surgery with emotional eating are more prone to having other psychiatric conditions, like depression or an anxiety disorder	88	1	Agree	83.0%
Patients with severe psychiatric conditions, like schizophrenia or bipolar disorder, should not undergo bariatric surgery, irrespective of whether the psychiatric condition is well controlled or not	91	1	Disagree	79.1%
Patients with depression and obesity who experience significant weight loss after bariatric surgery usually also experience improvement in their depressive symptoms	84	1	Agree	75.0%
Candidates for bariatric surgery with food addiction are more prone to having other psychiatric conditions, like depression or an anxiety disorder	88	1	Agree	73.9%
Overall, patients who have undergone bariatric surgery have an increased risk of suicide	79	2	Agree	70.9%
*Bariatric surgery increases the suicide rate among candidates for bariatric surgery who already have clinical depression*	*79*	*2*	*Agree*	*68.4%*
*Patients undergoing gastric bypass are more susceptible to developing problematic alcohol use post-operatively*	*79*	*2*	*Agree*	*57.0%*

Italicized statements did not reach consensus

*MBS* metabolic and bariatric surgery

*N = number of voters in the final/definitive round of voting on the statement

**Table 7:** Module 4—Patient preparation for metabolic and bariatric surgery (*N* = 94 voters in round 1; 79 in round 2)

Statements (*N* = 23)	N*	Rounds required	Most common selection	Percentage consensus
General health (*N* = 10)				
A comprehensive medical and nutritional evaluation should be completed before bariatric surgery	93	1	Agree	100.0%
Nutrient deficiencies should be evaluated and corrected in all candidates for bariatric surgery	93	1	Agree	98.9%
Among smokers, smoking cessation is recommended before bariatric surgery	93	1	Agree	96.8%
Sleep apnea screening is recommended, with testing only necessary in patients in whom there is a high suspicion of sleep apnea	92	1	Agree	89.1%
Weight reduction decreases a person’s future risk of developing cholangiocarcinoma	79	2	Not yet known	86.1%
Computed tomography or magnetic resonance imaging should be used routinely to screen for hepatocellular carcinoma in patients with metabolic-associated fatty liver disease	76	2	Disagree	81.6%
All antidiabetic drugs have an impact in reducing the risk of hepatocellular carcinoma in patients with metabolic-associated fatty liver disease	81	1	Disagree	80.2%
Pre-operative endoscopy should be performed in every patient undergoing bariatric surgery	88	1	Agree	76.5%
Screening for hepatocellular carcinoma should be performed in all patients with metabolic-associated fatty liver disease	76	2	Agree	71.1%
*There are differences between the different modes of weight reduction (calorie restriction, exercise, drugs, endoscopic and bariatric surgery) in terms of reducing the risk of hepatocellular carcinoma*	*77*	*2*	*Agree*	*66.2%*
COVID-19 (*N* = 13)				
Due to the increased risk of severe symptoms from COVID in patients with obesity, until the spread of COVID-19 is well controlled, bariatric surgery procedures should be reduced to a minimum to reduce the risk of viral exposure	79	2	Disagree	94.9%
Considering that patients with obesity are at higher risk of a severe COVID-19 course, more restrictive measures should generally be undertaken during hospitalization for bariatric procedures or related pre-operative evaluations	78	2	Agree	93.6%
Especially during the pandemic, metabolically sicker patients with obesity should be prioritized for bariatric surgery, since they are at greater risk from the pandemic and treatment decreases their risk	79	2	Agree	91.1%
Unvaccinated, metabolically-sicker patients with obesity should be prioritized for vaccination against COVID-19	89	1	Agree	87.6%
Unvaccinated or incompletely vaccinated patients scheduled for bariatric surgery who test negative for COVID-19 at admission can be placed in double rooms with other patients who have tested negative	79	2	Agree	83.5%
Since diabetes mellitus places patients at increased risk of a severe COVID-19 course, patients with diabetes or who are otherwise metabolically compromised warrant special protective measures during their care	78	2	Agree	83.3%
Outpatients undergoing pre-operative evaluations should have an antigenic COVID swab test on the day of the planned procedure or investigation	79	2	Agree	82.3%
Before gaining any kind of access to the hospital, all patients with obesity should be contacted by telephone and asked to report any recent potential COVID exposure or symptoms, as well as any situations or behaviors that might have placed them at particular risk of becoming infected	92	1	Agree	81.5%
Since vitamin D is thought to be a protective factor, measurement of and/or treatment with vitamin D should be considered prior to treating patients with obesity	90	1	Agree	80.0%
Since elevated interleukin-6 is considered a risk factor for a more severe COVID-19 course and is disproportionately elevated in patients with obesity, the level of IL-6 should be measured in all patients being treated for obesity, either before or at the beginning of their treatment	85	1	Disagree	76.5%
More stringent anticoagulation after surgery/endoscopy should be considered for patients undergoing MBS because of the increased risk of thrombosis due to obesity per se and COVID	76	2	Agree	76.3%
Patients scheduled for bariatric surgery who require hospitalization should have a PCR swab 24 h before hospital admission and, if their hospitalization is longer than 48 h, should have a second PSR swab at the time of hospital discharge	79	2	Agree	74.7%
Due to the increased risk of a severe COVID-19 course in patients with obesity, during the COVID-19 pandemic, patients undergoing bariatric surgery should be provided a single room, both pre- and post-operatively, throughout their hospitalization for surgery	78	2	Agree	70.5%

Italicized statements did not reach consensus

*MBS* metabolic and bariatric surgery, *COVID* coronavirus disease, *PCR* polymerase chain reaction test

*N = number of voters in the final/definitive round of voting on the statement,

**Table 8:** Module 5—Bariatric endoscopy (surgeons and endoscopists only; *N* = 58 voters in round 1; 54 in round 2)

Statements (*N* = 39)	N*	Rounds required	Most common selection	Percentage consensus
General statements (*N* = 5)				
Endoscopic bariatric and metabolic therapies include a diverse set of minimally invasive procedures that play unique and important roles in the treatment of obesity and related metabolic diseases and should be included as part of a multidisciplinary approach to managing these patients	58	1	Agree	98.3%
A prerequisite for any bariatric endoscopist should be endoscopic bariatric training, a curriculum still undefined, but which should include learning about the various surgical procedures, the physiology of obesity, and endoscopic skills	58	1	Agree	98.3%
Bariatric surgical centers should communicate a comprehensive care plan, both to patients and their primary care providers, including details about the surgical procedure, blood tests, required long-term vitamin supplements, and when patients need to be referred back	56	1	Agree	98.2%
There is currently inadequate empirical evidence to support the use of ANY bariatric endoscopic procedure as an option in multidisciplinary weight loss programs**	*54*	*1*	*Disagree*	*55.6%*
No bariatric endoscopic procedure is justified in patients with obesity whose only reason for weight loss is to look better.**	*54*	*1*	*Neither*	*50.0%*
Aspiration therapy (*N* = 8)				
Aspiration therapy *should be/should not be* considered for patients with class I obesity and obesity-related comorbidity	54	2	Should not be	90.7%
With aspiration therapy, replacements of the A-Tube and continued use will be necessary to achieve adequate long-term weight loss	53	2	Agree	86.8%
As an available option in multidisciplinary weight loss programs, there is currently enough empirical evidence to support the use of aspiration therapy	54	2	Disagree	85.2%
Aspiration therapy *should be/should not be* considered for patients with class 2 or 3 obesity	54	2	Should not be	85.2%
In patients with obesity whose only real reason for weight loss is to look better, it is reasonable to carefully consider aspiration therapy	58	1	Disagree	84.5%
The ability to induce meaningful weight loss and an acceptable risk profile are characteristics of aspiration therapy	54	2	Disagree	79.6%
Generating enough weight loss to induce improvement in obesity-related comorbidities is achievable with aspiration therapy	54	2	Disagree	75.9%
Aspiration therapy *should be/should not be* considered bridge therapy for patients with class 2 or 3 obesity in need of weight loss to improve outcomes for a specific surgery or medical treatment/ procedure (e.g., orthopedic surgery, organ transplant, fertility therapy, bariatric surgery)	54	2	Should not be	74.1%
Duodenal procedures (*N* = 2)				
As an available option in multidisciplinary weight loss programs, there is currently enough empirical evidence to support the use of duodenal mucosal resurfacing	58	1	Disagree	82.8%
As an available option in multidisciplinary weight loss programs, there is currently enough empirical evidence to support the use of a duodenal-jejunal bypass liner	58	1	Disagree	81.0%
Endoscopic gastric bypass revision (*N* = 5)				
Endoscopic gastric bypass revision with an endoscopic suturing device or plication device *should be/should not be* considered for patients with class 2 or 3 obesity and > 20% weight regain from a weight nadir after Roux-en-Y Gastric Bypass (RYGB)	53	2	Should be	79.2%
Endoscopic gastric bypass revision with an endoscopic suturing device or plication device *should be/should not be* considered for patients with > 20% weight regain from a weight nadir after Roux-en-Y gastric bypass (RYGB), regardless of their class of obesity at the time of weight regain	54	2	Should be	75.9%
In patients with obesity whose only real reason for weight loss is to look better, it is reasonable to carefully consider endoscopic gastric bypass revision with an endoscopic suturing or plication device	58	1	Disagree	72.4%
The ability to induce meaningful weight loss and an acceptable risk profile are characteristics of endoscopic gastric bypass revision with an endoscopic suturing or plication device	54	2	Disagree	70.4%
*Generating enough weight loss to induce improvement in obesity-related comorbidities is achievable with endoscopic gastric bypass revision with an endoscopic suturing device or plication device*	*54*	*2*	*Disagree*	*68.5%*
Endoscopic gastric plication (*N* = 7)				
Endoscopic gastric plication procedures *should be/should not be* considered in patients with class 3 obesity when they are not good surgical candidates or have declined surgery	54	2	Should be	87.0%
With endoscopic gastric plication procedures, adjunctive weight loss medications or repeat plication procedures may be necessary to achieve adequate long-term weight loss in some patients	58	1	Agree	86.2%
Endoscopic gastric plication procedures *should be/should not be* considered for patients who are in the overweight category and have obesity-related comorbidities	53	2	Should be	83.0%
In patients with obesity whose only real reason for weight loss is to look better, it is reasonable to carefully consider endoscopic gastric plication procedures, like POSE	53	2	Disagree	81.1%
*The ability to induce meaningful weight loss and an acceptable risk profile are characteristics of endoscopic gastric plication procedures, like POSE*	*53*	*2*	*Agree*	*62.3%*
*As an available option in multidisciplinary weight loss programs, there is currently enough empirical evidence to support the use of endoscopic gastric plication procedures, like POSE*	*53*	*2*	*Agree*	*56.6%*
*Generating enough weight loss to induce improvement in obesity-related comorbidities is achievable with endoscopic gastric plication procedures, like POSE*	*53*	*2*	*Agree*	*56.6%*
Endoscopic gastric suturing (*N* = 4)				
With endoscopic gastric suturing procedures, adjunctive weight loss medications or repeat procedures may be necessary to achieve adequate long-term weight loss in some patients	54	1	Agree	88.9%
Endoscopic gastric suturing procedures *should be/should not be* considered for patients who are in the overweight category and have obesity-related comorbidities	54	2	Should be	85.2%
Endoscopic gastric suturing procedures *should be/should not be* considered in patients with Class 3 obesity when they are not good surgical candidates or have declined surgery	55	1	Should be	72.7%
*In patients with unsatisfactory weight loss after an endoscopic sleeve gastroplasty (ESG) procedure, endoscopic treatment can be repeated at most once, more than once, or not at all (in lieu of surgical revision)*	*53*	*2*	*Not at all*	*57.4%*
Intragastric balloons (*N* = 8)				
With intragastric balloons, adjunctive weight loss medications or repeat balloon placements may be necessary to achieve adequate long-term weight loss in many patients	58	1	Agree	87.9%
The ability to induce meaningful weight loss and an acceptable risk profile are characteristics of intragastric balloons	54	2	Agree	85.2%
Intragastric balloons *should be/should not be* considered for patients with class 1 or 2 obesity	58	1	Should be	82.8%
As an available option in multidisciplinary weight loss programs, there is currently enough empirical evidence to support the use of intragastric balloons	58	1	Agree	81.0%
Intragastric balloons *should be/should not be* considered bridge therapies for patients with class 2 or 3 obesity in need of weight loss to improve outcomes for a specific surgery or medical treatment/procedure (e.g., orthopedic surgery, organ transplant, fertility, bariatric surgery)	58	1	Should be	81.0%
Intragastric balloons *should be/should not be* considered for patients who are in the overweight category and have obesity-related comorbidities	57	1	Should be	80.7%
In patients with obesity whose only real reason for weight loss is to look better, it is reasonable to carefully consider intragastric balloons	54	2	Agree	72.2%
*Generating enough weight loss to induce improvement in obesity-related comorbidities is achievable with intragastric balloons*	*53*	*2*	*Agree*	*62.3%*

Italicized statements did not reach consensus

*ESG* endoscopic sleeve gastroplasty, *POSE* primary obesity surgery using an endoluminal approach

*N = number of voters in the final/definitive round of voting on the statement

**New statement added in round 2 to clarify round 1 responses

**Table 9:** Module 6—Outcomes and follow-up (*N* = 94 voters in round 1; 79 in round 2)

Statements (*N* = 32)	N*	Rounds required	Most common selection	Percentage consensus
Some degree of weight regain is normal between 2 and 10 years after MBS	90	1	Agree	100.0%
Significant weight regain, as well as the presence of obesity-related medical problems, may require further medical, endoscopic, or surgical treatment after MBS	88	1	Agree	100.0%
After bariatric surgery, annual follow-up is recommended life-long	90	1	Agree	100.0%
Bariatric surgical centers should work jointly with primary care providers to provide follow-up and access to appropriate healthcare professionals, as clinically indicated	90	1	Agree	100.0%
After MBS, if a patient still has severe obesity with obesity-related medical problems 2 years after MBS, additional therapy may be indicated (medical, endoscopic, or surgical)	89	1	Agree	98.9%
Follow-up after endoscopic bariatric treatment must always include nutrition counselling	90	1	Agree	98.9%
Bone health should be evaluated in the postoperative period, especially in individuals considered at high risk for osteoporosis	89	1	Agree	98.9%
Substantial net *health benefits* may be anticipated, on a societal level, from the wider use of bariatric surgical procedures in patients with severe obesity	88	1	Agree	98.9%
Since severe obesity shows strong socioeconomic patterning, bariatric surgery has the potential to reduce obesity-related inequalities in health, as long as there is equitable patient selection	89	1	Agree	98.9%
Patients presenting with significant weight regain after MBS require an extensive evaluation, including anatomic studies (e.g., EGD) and evaluation by the multidisciplinary team	89	1	Agree	97.8%
Weight regain after MBS is multi-factorial, potentially including nutritional non-compliance, physical inactivity, mental health issues, and anatomical issues encountered during surgery	91	1	Agree	96.7%
Relative to medical therapy, in patients with obesity and type 2 diabetes, bariatric surgery is generally, in the long run…	89	1	Agree	95.5%
Patients presenting with GERD symptoms, with or without weight regain after MBS, require an objective assessment for GERD, including pH studies with or without manometry	87	1	Agree	95.4%
Substantial net *economic benefits* may be anticipated, on a societal level, from the wider use of bariatric surgical procedures in patients with severe obesity	87	1	Agree	95.4%
In patients undergoing MBS who experience unsatisfactory post-op weight loss, supplementary medical treatment (e.g., glucagon-like peptide-1 agonist) should be added as combination therapy	89	1	Agree	93.3%
There is no uniformly recognized definition for what constitutes significant weight regain after MBS	90	1	Agree	88.9%
Follow-up after endoscopic bariatric treatment must always involve a complete multidisciplinary team (e.g., dietitian or nutritionist, psychologist, exercise therapist)	89	1	Agree	88.8%
Different definitions of MBS success include achieving > 50% EWL, a BMI < 35 kg/m^2^, and > 10% TWL%	89	1	Agree	86.5%
The cost benefit of bariatric surgery is greater in patients with obesity-related comorbidity, greater in patients with no obesity-related comorbidity, or about the same on these two populations	88	1	Greater with comorbidity	86.4%
Similar cost-effectiveness may be anticipated in diverse groups undergoing MBS, including men and women, patients across a wide range of ages, and patients with different levels of social deprivation	78	2	Agree	85.9%
Increasing patient selection for bariatric surgery to include patients who are less obese will increase the overall societal health benefits of bariatric surgery	78	2	Agree	85.9%
There is no uniformly recognized definition for what constitutes surgical success after MBS	89	1	Agree	80.9%
Due to the increased risks of surgery in those who are more obese, in patients who are very obese, bariatric surgery is less cost effective than in those who are less obese	88	1	Disagree	80.7%
The cost benefit of bariatric surgery is greater in younger than older patients, greater in older than younger patients, or about the same in youths and seniors	79	2	Greater in younger	79.7%
The most commonly used definition for significant weight regain after MBS is achieving less than 50% EWL	79	2	Agree	78.5%
All forms of bariatric surgery are effective, overall, at improving patients’ quality of life	90	1	Agree	77.8%
Patients with a BMI between 40 and 50 kg/m^2^ experience the greatest cost benefit from bariatric surgery	85	1	Agree	77.6%
Weight regain tends to be greater in patients with super obesity (BMI > 50 kg/m^2^)	84	1	Agree	76.2%
Weight regain depends on the type of MBS performed	88	1	Agree	72.7%
Weight regain after MBS, even when significant, should never be called failure	89	1	Agree	71.9%
*The cost effectiveness of bariatric surgery is lost if patients regain all the weight they lost post-operatively within the next 5–10 years*	*78*	*2*	*Agree*	*67.9%*
*For the 1st year after endoscopic bariatric treatment, some member of a patient’s obesity-management team should see them to evaluate their overall response to treatment and identify complications*	*79*	*2*	*At least monthly*	*57.5%*

Shaded statements did not reach consensus

*MBS* metabolic and bariatric surgery, *BMI* body mass index, *EWL* excess weight loss, *TWL* total weight loss, *EGD* esophagogastroduodenoscopy

*N = number of voters in the final/definitive round of voting on the statement

On epidemiology and risk factors, unanimous consensus was reached that all medical societies must address obesity systematically and that regular longitudinal national and regional surveillance is necessary. Strong consensus was achieved defining obesity as a chronic disease that increases both morbidity and mortality risks; that emotional eating is a common feature but also that eating binges not universal among those with obesity; and that ethnicity and geographical factors are important, both pathophysiologically and when considering interventions. Experts agreed that food addiction is a valid clinical entity, and common among patients undergoing MBS, especially those with problematic alcohol and/or drug use; but were split on whether food addiction affects a great majority of patients considering MBS. They also agreed that binge eating is a risk factor for weight regain after MBS, but disagreed it is a risk factor for suicidal ideations/attempts. All Module 1 results are summarized in Table 3.

On patient selection (Table 4), there was 100% consensus that global rates of obesity are increasing in children and adolescents; that obesity during childhood or adolescence portends obesity in adulthood; that severe obesity in the young portends significant obesity-related co-morbidity, like diabetes and hypertension; that MBS in youths requires a multi-disciplinary team with experience dealing with youths and their families; and that inadequate public and physician knowledge and scarce long-term results of MBS in youths are barriers to MBS use in youths. There also was near-unanimous agreement that life-long monitoring is necessary for youths who undergo MBS and that MBS in youths should be performed by experienced bariatric surgeons with a proven track record of success in adults. Experts agreed that enough empirical evidence has been published supporting MBS as the most effective therapy for severe obesity in youths and that MBS outcomes in youths are similar to those achieved in adults. However, certain MBS procedures, like biliopancreatic diversion (BD) and one-anastomosis gastric bypass (OAGB), were not recommended for youths.

Considering seniors, there again was consensus that MBS is generally effective and safe and increases QoL and that age should not be the only consideration when deciding on surgery. Conversely, there was consensus that operating time is directly predictive of negative outcomes in seniors, and that seniors’ risks from MBS are greater than adolescents. No consensus was reached concerning on the age when operative candidates should be considered elderly, on outcomes post-Roux-en-Y gastric bypass (RYGB) and laparoscopic sleeve gastrectomy (LSG) relative to outcomes in adolescents, or on the gold standard MBS procedure for seniors. Table 5 ranks 23 pre-operative factors by their relative level of importance, with all but financial means and thyroid disease considered very important by ≥ 70% of our experts.

Among psychological issues, there was consensus disagreement that patients undergoing MBS always develop problematic alcohol use or mostly experience worsened depression post-operatively. Experts also disagreed that those patients with pre-MBS cognitive depressive symptoms usually do not improve post-operatively, as opposed to those who have meaningful post-operative weight loss and usually experience improvement in their depression post MBS. However, there also was consensus agreement that suicide is more common in patients who have undergone MBS. Strong consensus was reached that a comprehensive psychological evaluation is necessary pre-operatively, and that even patients with severe psychiatric illness can undergo MBS if it is well controlled. Experts also agreed that patients with food addiction are more likely to have other psychiatric conditions—like depression and anxiety—than those without, and that cognitive behavioral therapy is the best therapeutic strategy for patients at high risk of binge eating. Further results on psychological issues are summarized in Table 6.

For preparatory steps prior to MBS, consensus was reached on the need for comprehensive medical and nutritional evaluations, identifying and correcting all nutritional deficiencies, smoking cessation, and pre-operative endoscopy, with sleep apnea screening only necessary in those considered at high risk. Experts disagreed that routine CT or MRI is required to screen for hepatocellular carcinoma prior to MBS and that all anti-diabetic drugs reduce the risk of this cancer in patients with non-alcoholic fatty liver disease (NAFLD). Table 7 summarizes further results, including anti-COVID 19 steps to take prior to MBS.

Among the 58 experts who performed endoscopic metabolic and bariatric therapy (EMBT), almost unanimous consensus was reached on the unique and important roles these procedures have managing obesity; that adequate endoscopic bariatric training is required for practitioners; and that MBS centers should communicate a comprehensive care plan to patients and their primary care providers, including testing, supplements, and when to be referred back for re-evaluation. Table 8 also specifically summarizes consensus opinions on aspiration therapy, duodenal procedures, endoscopic gastric bypass, gastric plication and suturing procedures, and intragastric balloons (IGBs). Among these, the greatest support was expressed for IGB and least for aspiration therapy and duodenal bypass, with intermediate support expressed for gastric procedures involving bypass, plication, or suturing, depending on the situation. The only procedures for which currently published empirical evidence was considered adequately supportive for them to no longer be considered of uncertain efficacy were those involving balloons. Intragastric balloons also were the only procedures considered acceptable for the sole purpose of helping patients “look better” and were voted acceptable “bridge therapy” for patients scheduled for later MBS.

Regarding post-procedural follow-up and outcomes, unanimous consensus was expressed that some degree of weight regain is normal 2–10 years after MBS, but also that appreciable weight regain may require further medical, endoscopic, or surgical treatment. Experts also unanimously agreed that post-MBS follow-up should be lifelong and that MBS centers should work jointly with patients' primary care providers to provide follow-up and access to appropriate healthcare professionals, as indicated. Near-unanimous agreement was expressed on the potential need for further treatment in patients with continued severe obesity and obesity-related problems two years after MBS, and on the need for comprehensive multi-disciplinary assessments in patients experiencing appreciable post-operative weight regain. Unsatisfactory post-operative weight loss was also considered an indication for supplementary anti-obesity medication (AOM). However, 93.3% and 80.9% agreed, respectively, that no uniformly-recognized definitions exist for either “significant weight regain” or “surgical success.”

For follow-up, nutrition counselling was considered an essential component of post endoscopic treatment by 98.9%, while assessing bone health and ruling out gastroesophageal dysfunction were considered important in patients deemed at high risk for osteoporosis and gastroesophageal reflux disease (GERD), respectively. Consensus agreement also was achieved on several statements pertaining to the benefits of MBS at a societal level. Further results are summarized in Table 9.

## Discussion

Clinical management of people with obesity has evolved tremendously over the past decade as understanding of this chronic disease has improved. Such advances include more universal acceptance of obesity as a disease. Despite this, its prevalence continues to rise worldwide in all age groups [2–4] as is its economic burden on healthcare systems [32]. In addition, the percentage of patients seeking any form of effective therapy for their obesity remains very low. There is widespread agreement, even beyond the current panel of experts, that a dire need exists to alter obesity’s current world trajectory and find ways to both prevent and treat it in more individuals. Two options that achieved unanimous consensus in our expert panel might achieve both goals: first, for all medical societies to cooperate to address the problem systematically; and second, for longitudinal surveillance to be conducted routinely at both regional and national levels. Two examples of multinational obesity surveillance programs that have generated useful data are the Scandinavian Obesity Registry (SOReg) [33] and German Bariatric Surgery Registry [34], the latter having existed for > 60 years. Such data have generated publications on crucial issues like short-term and long-term outcomes after MBS and a 10-year post-operative mortality rate of just 0.06% over the first 90 post-operative days, as well as data on immediate and longer-term post-operative complications, weight loss, comorbidity management, impact of patient age on outcomes, and comparing different MBS procedures [33–41]. Though such data are tremendously valuable, only a very small percentage of individuals with obesity ever undergo MBS, and it is the remaining huge majority for which closer surveillance remains necessary. More realistic, perhaps, are physician and public obesity education campaigns to increase awareness both about the health hazards associated with obesity (e.g., increased risk of cancer), and the need for comprehensive, multidisciplinary treatment, especially for those whose obesity has become severe and/or having obesity-associated comorbidities.

Another issue on which unanimous consensus was repeatedly reached was obesity in children and adolescents, all our experts agreeing that global rates of obesity are currently increasing in youths and that most youths with obesity continue to have obesity in adulthood. Additionally, youths with severe obesity are at risk of significant obesity-related comorbidities like diabetes. Unanimity also was expressed that MBS in adolescents requires an experienced, multi-disciplinary team with experience dealing with youths and their families, and that inadequate physician and public awareness and insufficient long-term outcome data are barriers against the referral of adolescents who might benefit from MBS. Pertaining to insufficient data, five meta-analyses documenting the beneficial effects of MBS in adolescents (including sustained weight reduction, improvements in some obesity-related comorbidities, and improved QoL) have been published [42–46]. However, few studies have had follow-up beyond five years and virtually none followed youths into adulthood. Data also are scant on potential nutritional and developmental difficulties [46].

In our survey, unanimous consensus was reached on five additional statements, all pertaining to surgical treatment or post-surgical follow-up. Unanimously expressed opinions were that multidisciplinary assessment is necessary prior to MBS; that some degree of weight regain is normal from 2 to 10 years after MBS; that significant weight regain, or the presence/persistence of obesity-related medical problems may require further medical, endoscopic, or surgical treatment; that follow-up after MBS should be lifelong; and that MBS centers should work jointly with their patients’ primary healthcare providers to ensure adequate follow-up and access to other healthcare professionals. Regarding MBS patient selection, the pre-operative factors rated very important by almost all experts were the patient’s overall level of health and fitness, presence and/or nature of comorbid illness, cognitive ability to understand the procedure and instructions, and presence of either alcohol or another substance abuse.

Repeatedly expressed was the need for multiple healthcare practitioners spanning different disciplines, especially for patients considering MBS. This should begin with a multi-disciplinary pre-operative assessment to determine each patient’s eligibility. Such assessments also are necessary to identify co-morbid medical, nutritional, and psychological disorders and barriers to treatment success and attempt to address as many of these barriers pre-operatively as possible. Also necessary is to otherwise prepare patients for surgery, including educating them concerning realistic goals, potential post-operative symptoms, high likelihood of some weight regain or other set-backs, and vital importance of continued, life-long follow-up. This multimodal management requires collaboration from members of a multidisciplinary team that includes dieticians/nutritionists, behavioral therapists, physicians, endocrinologists, endoscopists, and surgeons.

Post-operatively, patients continue to require ongoing, multi-disciplinary care to manage their weight loss program and obesity-associated comorbidities. They also require monitoring for the life-altering effects of surgery, like the risk of potentially catastrophic nutritional deficiencies that may vary depending on the specific MBS performed [22, 47]. Each patient’s psychological state must also be followed, given recent data suggesting a slightly elevated risk of suicide in both adolescents and adults who undergo MBS [48, 49]. Potential contributory factors include forced alterations in foods they can and cannot eat, gastrointestinal symptoms secondary to food intolerance, and unrealized, unrealistic expectations about the extent of weight loss they may experience post-operatively, leading to depression, anxiety, reduced sense of self-worth, and other forms of psychological distress. Monitoring also is essential to detect the re-emergence of detrimental eating patterns, like binge eating, as such factors may predict poorer post-operative weight management [50].

Every expert consensus survey has the potential for bias, given that participants may already have a predilection to utilize a particular practice to have become experts in its use. In addition to adopting the Delphi approach (characterized by voter anonymity, largely eliminating acquiescence bias), our survey was unique in that we sought the opinions of a uniquely-broad array of healthcare practitioners that included surgeons, non-surgical physicians, and non-physician experts in nutrition and psychological counselling. All participants were invited to vote on any statement with which they felt comfortable, except for one module on endoscopic therapy restricted to surgeons and endoscopists. Recognizing worldwide differences in obesity management, we also included experts from every permanently inhabited continent. In this manner, we attempted to minimize the widely held criticisms of consensus-survey critics of “like-minded individuals voting together.” We further worded survey statements so a sizeable majority neither favored nor opposed the concept/belief/approach presented, with the remaining statements evenly balanced between favorable and unfavorable. The order of response options also was altered so the most favorable option was listed anywhere from first to last.

We nonetheless acknowledge that consensus surveys are level V evidence, and based upon opinions, rather than experimentally-generated data. That said, all our voters were widely renowned experts in obesity management and, thus, both familiar with such research and particularly qualified to interpret it. In other words, their opinions were based not just upon their extensive experience, but on their expansive knowledge of relevant research. Moreover, as stated initially, this consensus survey was conducted to aid in generating joint IFSO-WGO guidelines, for which over 1000 scientific references have also been utilized to frame the discussion. The consensus opinions we sought to aid in drafting those guidelines were for issues for which existing literature is either non-definitive—requiring appreciable interpretation—or largely lacking, especially on issues that might be particularly difficult to study empirically, like whether EMBT can be justified for aesthetic purposes only.

Since the conclusion of this joint IFSO-WGO Delphi Survey, 2022 ASMBS/IFSO Guidelines on Indications for Metabolic and Bariatric Surgery have been published, and many of those guidelines support our survey results [51].
